# Larger hippocampal dimensions in meditation practitioners: differential effects in women and men

**DOI:** 10.3389/fpsyg.2015.00186

**Published:** 2015-03-06

**Authors:** Eileen Luders, Paul M. Thompson, Florian Kurth

**Affiliations:** ^1^Department of Neurology, David Geffen School of Medicine, University of California at Los Angeles, Los Angeles, CA, USA; ^2^Imaging Genetics Center, Institute for Neuroimaging and Informatics, Keck School of Medicine, University of Southern California, Los Angeles, CA, USA

**Keywords:** brain, gender, hippocampus, meditating, mindfulness, MRI, sex

## Abstract

On average, the human hippocampus shows structural differences between meditators and non-meditators as well as between men and women. However, there is a lack of research exploring possible sex effects on hippocampal anatomy in the framework of meditation. Thus, we obtained high-resolution magnetic resonance imaging data from 30 long-term meditation practitioners (15 men/15 women) and 30 well-matched control subjects (15 men/15 women) to assess if hippocampus-specific effects manifest differently in male and female brains. Hippocampal dimensions were enlarged both in male and in female meditators when compared to sex- and age-matched controls. However, meditation effects differed between men and women in magnitude, laterality, and location on the hippocampal surface. Such sex-divergent findings may be due to genetic (innate) or acquired differences between male and female brains in the areas involved in meditation and/or suggest that male and female hippocampi are differently receptive to mindfulness practices.

## INTRODUCTION

Meditation-specific features of the hippocampus, including its connecting fiber tracts, have been examined using different imaging modalities, such as magnetic resonance imaging (MRI) and diffusion tensor imaging (DTI). Outcomes of these imaging studies point to greater hippocampal dimensions in meditators, such as bigger hippocampal volumes, larger hippocampal distances, more hippocampal gray matter (GM), as well as a higher fractional anisotropy in fibers connecting to the hippocampus (Holzel et al., 2008; Luders et al., 2009b, 2011, 2013a,b; Murakami et al., 2012; Leung et al., 2013). Moreover, these cross-sectional findings are complemented by outcomes from longitudinal analyses suggesting an increase of hippocampal GM as a result of meditating (Holzel et al., 2011).

This solid body of literature on hippocampus-specific meditation effects is matched by an at least equally significant body of literature on hippocampus-specific differences between male and female brains. For example, there are reports of sex differences in hippocampal anatomy (Filipek et al., 1994; Goldstein et al., 2001; Szabo et al., 2003; Mouiha and Duchesne, 2011; Han et al., 2013; Perlaki et al., 2014; Persson et al., 2014), hippocampal function (Mackiewicz et al., 2006; Goldstein et al., 2010), hippocampal development (Benes et al., 1994; Giedd et al., 1996; Suzuki et al., 2005; Hu et al., 2013; Lin et al., 2013) as well as hippocampal pathology and age-related atrophy (Murphy et al., 1996; Briellmann et al., 2000; Exner et al., 2008; Li et al., 2014).

The aforementioned meditation effects and sex differences within the hippocampus raise the question of whether hippocampus-specific meditation effects manifest differently in male and female brains. The lack of research addressing that question might be due to the rather small number of subjects included in many meditation studies and/or the unequal distribution of male and female subjects. We therefore conducted the present study leveraging an existing data set consisting of 15 male and 15 female meditators as well as 15 male and 15 female control subjects. Specifically, we set out to investigate the modulating effects of biological sex on hippocampal anatomy in the framework of meditation. For this purpose, we combined a traditional volumetric approach (assessing global hippocampal volumes) with a modern surface-based mapping technique (assessing local hippocampal distances) and tested for significant group-by-sex interactions followed by mapping meditation effects in men and women separately.

## MATERIALS AND METHODS

### SUBJECTS

The study included 30 meditators and 30 control subjects, where the sample was identical to the one analyzed previously (Luders et al., 2012b, 2013b). Meditators were recruited by distributing study flyers at meditation centers, by postings on center-specific e-mail lists, or by word of mouth through meditators who had already participated in our study. Interested subjects contacted the lab and were subsequently pre-screened for eligibility via e-mail or phone. Subjects who met study inclusion criteria were scheduled for a 2-h appointment on the University of California, Los Angeles (UCLA) campus (for details on subject-specific meditation styles and practices, refer to Luders et al., 2012b). The final composition of the meditation sample ultimately determined the composition of the control sample. More specifically, for each meditator, we selected one control subject from an existing database^1^ aiming at the closest pair-wise match with respect to sex, handedness, and age. The maximum allowed age difference within a sex-matched pair was 2 years. Criteria for eligibility and procedures to screen the control subjects are detailed elsewhere (Mazziotta et al., 2009).

The two resulting samples (meditators/controls) each contained 15 males and 15 females and consisted of 28 right-handers and two left-handers (all left-handers were males). Handedness was determined based on preferences for selected activities, such as writing, throwing, holding, opening, etc., using a modified version of the Edinburgh Inventory (Oldfield, 1971). Age ranged from 24 to 64 years, with a mean age of 47.3 years for meditators and also 47.3 years for controls (SD: ± 11.7 and 11.8, respectively). Within the meditation sample, years of meditation practice ranged from 5 to 46 years, with a mean practice duration of 20.2 years (SD: ± 12.2 years). The practice duration did not differ significantly between male meditators (mean ± SD: 19.9 ± 11.5 years) and female meditators (mean ± SD: 20.5 ± 13.3 years). All subjects gave informed consent according to institutional guidelines and the study was approved by the Institutional Review Board of the UCLA.

### DATA ACQUISITION AND IMAGE PREPROCESSING

All subjects (meditators/controls) were scanned on the same site, using the same scanner, and following the same scanning protocol. Specifically, magnetic resonance images were acquired on a 1.5 Tesla Siemens Sonata scanner (Erlangen, Germany) using an 8-channel head coil and a T1-weighted MPRAGE sequence (1900 ms TR, 4.38 ms TE, 15° flip angle, 160 contiguous sagittal slices, 256 mm × 256 mm FOV, 1 mm × 1 mm × 1 mm voxel). The obtained structural brain images were then corrected for intensity inhomogeneities and linearly transferred into a standard space using six-parameter (rigid-body) normalizations, as previously detailed (Luders et al., 2013b).

### TOTAL BRAIN VOLUME MEASURES

Prior to our hippocampal analyses, we set out to address if male meditators and male controls (female meditators and female controls, respectively) differ in total brain volume. For this purpose, all image volumes were tissue-classified into GM, white matter (WM), and cerebrospinal fluid (CSF) using SPM8^2^ and the VBM8 toolbox^3^, as described elsewhere (Luders et al., 2009a). Tissue volumes were determined based on the respective tissue-classified partitions (i.e., GM, WM, and CSF) in native space. Total brain volume was calculated (in ml) by adding GM, WM, and CSF volumes. Male meditators and male controls did not show significant differences in total brain volume (mean ± SD: 1514.02 ± 111.96 versus 1514.93 ± 111.12), and neither did female meditators relative to female controls (1378.03 ± 112.49 versus 1360.08 ± 99.13).

### HIPPOCAMPUS ANALYSES

#### Labeling and reliability

The hippocampus was labeled manually in contiguous coronal brain sections, as previously described (Luders et al., 2013b). To determine intra-rater reliability, the hippocampus was labeled twice, by the same rater, in five randomly selected brains revealing intra-class correlations for hippocampal volume of *r_I_* = 0.95. In addition, the hippocampus was labeled five times, by the same rater, within one randomly selected brain revealing a volumetric overlap of 85% for all labels. The overlap was defined as the volume of the intersection of the five labels, divided by the mean volume of these labels, multiplied by 100.

#### Global measures

Global left and right hippocampal volumes were established (in mm^3^) based on the dimensions and number of voxels constituting the hippocampal labels. Left and right global hippocampus measures were statistically compared between groups defined by meditation status (meditators/controls) and biological sex (men/women). More specifically, we used a general linear model with the left and right hippocampal values as dependent variables, group as fixed factor, and sex as covariate. As a safeguard against type I error, Bonferroni corrections were applied using a threshold of *p* ≤ 0.025 to account for the two (left/right) dependent variables. A significant group-by-sex interaction was followed by *post hoc* comparisons within men and women, separately.

#### Local measures

First, the manually outlined hippocampal labels (described above) were converted into three-dimensional shape representations of the left and right hippocampus. Then, parametric surface meshes (Thompson et al., 1996a,b) were generated automatically, precisely following the outer contours of the hippocampal shapes. As described previously (Luders et al., 2013b), these parametric surface meshes “resemble a gridded surface of equally spaced points, where the array of these points is standardized across all subjects establishing a point-by-point correspondence.” For each left and right hippocampal mesh, a medial curve was defined along the long axis of the hippocampus threading down the hippocampal center. The radial distances (in mm) from this medial curve to each hippocampal surface point were measured and subsequently used in the statistical analysis. For more methodological details, including illustrations of the surface mesh modeling and radial distance mapping, please refer to a previous publication (Thompson et al., 2004). The local hippocampal distances were compared between groups using the general linear model, as detailed above for the global analyses. However, to explore differential effects across the hippocampal surface, the exact locations of significant group-by-sex interactions as well as any *post hoc* effects were mapped using uncorrected thresholds at *p* ≤ 0.05.

## RESULTS

### GLOBAL MEASURES

Descriptively, left and right hippocampal volumes were larger, on average, in male meditators compared to male controls; they were also larger in female meditators compared to female controls (see Table 1). The group-by-sex interaction was significant for the left hippocampus (*p* = 0.002) but not for the right hippocampus (*p* = 0.46). Conducting *post hoc* comparisons separately within males and females, left hippocampal volumes were significantly larger in male meditators than male controls (*p* = 0.02) as well as in female meditators than female controls (*p* = 0.046). Significant meditation effects with respect to right hippocampal volumes were not detectable in males (*p* = 0.722) or in females (*p* = 0.291).

**TABLE 1:** **Hippocampal volumes (mean ± SD) in mm^3^**.

**Left hippocampus**		**Right hippocampus**	
Male meditators	Male controls	Male meditators	Male controls
3638.20 ± 316.85	3346.00 ± 333.23	3600.87 ± 436.05	3533.93 ± 593.56
			
Female meditators	Female controls	Female meditators	Female controls
3404.73 ± 311.18	3192.80 ± 239.73	3568.40 ± 381.43	3397.73 ± 480.72

### LOCAL MEASURES

**FIGURE 1:**
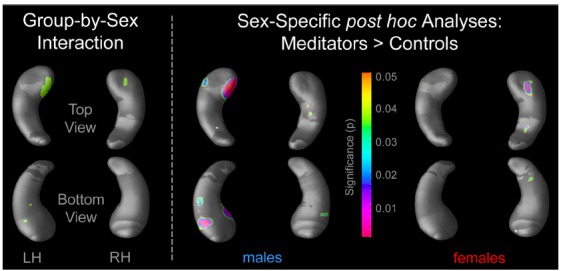
**Significant hippocampal differences.** The left panel depicts significant group-by-sex interactions (*p* ≤ 0.05). The right panel illustrates where *post hoc* analyses revealed significant group differences (meditators > controls), separately within males and females. The color bar encodes the significance (*p*) of the group differences. Hippocampal regions in gray indicate where no significant group differences were observed. LH, left hippocampus; RH, right hippocampus.

There were significant group-by-sex interactions, both within the left and right hippocampus (Figure 1, left). Conducting *post hoc* comparisons within males, radial distances were significantly larger in male meditators than in male controls. Significance clusters were evident in both hemispheres but stronger within the left hippocampus, with most pronounced effects in the hippocampal head (Figure 1, middle). Conducting *post hoc* comparisons within females also revealed significantly larger radial distances in female meditators than in female controls. However, in contrast to the laterality effect observed within males, significance clusters within females were almost exclusively detectable in the right hippocampus (Figure 1, right). Neither within males nor within females was there any hippocampal region where controls had significantly larger radial distances than meditators.

## DISCUSSION

To our knowledge, this is the first study examining potential modulating effects of biological sex on hippocampal anatomy in the framework of meditation. Our analyses were applied in a well-matched sample of 30 meditators (15 men/15 women) and 30 controls (15 men/15 women), where meditators had, on average, more than 20 years of experience (with a minimum of 5 years), thus constituting true long-term practitioners. In accordance with the outcomes of our previous study of meditation effects on hippocampal anatomy by pooling male and female brains together (Luders et al., 2013b), we observed that hippocampal dimensions were enlarged both in male and in female meditators when compared to sex- and age-matched controls. In addition, our current analyses revealed that meditation effects, albeit present in both sexes, differ between men and women in terms of the magnitude of the effects, the laterality of the effects, and the exact location of the effects detectable on the hippocampal surface.

The observed group-by-sex interactions and sex-divergent effects are intriguing and perhaps reflective of differential (innate) conditions in male and female brains, as also implied by the large number of reports on sex differences within the hippocampus (see Introduction). At the same time, it is possible that male and female meditators may require (or employ) different amounts or elements of practice to experience desired effects. Both possibilities, independently or interacting with each other, might be accompanied by a sex-specific engagement of certain hippocampal subsections during meditation. Such a sex-specific hippocampal engagement might result in a sex-specific impact on hippocampal anatomy, where additional effects may manifest if male and female hippocampi are differently susceptive to the practice. Future research is clearly necessary, not only to uncover the rather complex underlying mechanisms but also answer the age-old question if meditation induces (sex-specific) brain changes or if a (sex-specific) unique brain anatomy preceded the meditation practice, as further discussed elsewhere (Luders et al., 2012a, 2013b). Both options are likely and not necessarily mutually exclusive, at least with respect to the hippocampus: biological sex as well as (sex-specific) experiences seem to play an integral role, not only in activating hippocampal functions but also shaping hippocampal structure, including adult hippocampal neurogenesis (Mitsushima, 2010; Galea et al., 2013). However, at this point, given the cross-sectional nature of our study, definite conclusions on the causality of the observed group-by-sex interactions and sex-specific group differences are not warranted.

### CORRESPONDENCE WITH PREVIOUS STUDIES

Our current observations are in line with prior reports of sex differences in hippocampal function, development, atrophy and pathology (Benes et al., 1994; Giedd et al., 1996; Murphy et al., 1996; Briellmann et al., 2000; Suzuki et al., 2005; Mackiewicz et al., 2006; Exner et al., 2008; Goldstein et al., 2010; Hu et al., 2013; Lin et al., 2013; Li et al., 2014), which in turn may be linked to sex differences in hippocampal anatomy (Filipek et al., 1994; Goldstein et al., 2001; Szabo et al., 2003; Mouiha and Duchesne, 2011; Han et al., 2013; Perlaki et al., 2014). Moreover, our current observations are in line with prior reports of altered hippocampal features in meditators compared to controls (Holzel et al., 2008; Murakami et al., 2012; Leung et al., 2013).

Although existing mindfulness research seems to lack sex-specific analyses—at least with respect to addressing brain anatomy—the observed group-by-sex interactions seem to be in accordance with a recent study reporting sex-divergent outcomes when assessing the impact of a mindfulness intervention on behavioral measures/psychological constructs (de Vibe et al., 2013). More specifically, administering a 7-week mindfulness-based stress reduction (MBSR) program, that study detected significant changes in mental distress, study stress and well-being in female students but not in male students. Contrasting such lack of an effect in one sex, the current study observed meditation effects in both sexes (meditators > controls). So, at first sight, there seems to be a partial divergence between the outcomes of the aforementioned study (de Vibe et al., 2013) and our current observation, as one might expect a lack of meditation effects on hippocampal anatomy in males. However, de Vibe et al. (2013) also emphasize that males did experience a small effect on mental distress but that “this effect was not statistically significant, possibly due to the fact that there were significantly fewer men in the intervention group than the control group.” Furthermore, given that 118 women but only 26 men received the MBSR intervention in de Vibe’s study, the resulting statistical power (i.e., disadvantageous in men) might have caused the lack of significance within men. Since our study was gender-balanced with equal morphometric variance across groups (i.e., affording the same statistical power within men and women) this may explain why meditation effects were observed in both sexes. Nevertheless, the nature of the effect was still different in male and female meditators, which seems to agree with de Vibe’s findings. Given the lack of other publications in this specific field, it seems tempting to (overly) relate both studies and find plausible explanations for convergences and divergences. However, as both studies are extremely different in many respects—not only in ages of the subjects, type of meditation, and duration of practice but also the actual study design and outcome measures—caution is warranted to not over-interpret apparent correspondences and discrepancies. Follow-up studies using more highly powered samples and gender-balanced designs are clearly necessary to further expand this currently understudied field of research.

### IMPLICATIONS FOR FUTURE RESEARCH

Importantly, the hippocampus is not a single homogeneous structure but regionally segregated by architecture, connectivity, and functional relevance (Amunts et al., 2005). Our refined local measures (i.e., radial hippocampal distances) already provide substantial additional detail beyond more conventional global measures (i.e., hippocampal volumes), which ultimately were sufficient to uncover differential meditation effects across the hippocampal surface and the sex-specific significance profiles. However, future studies with methods that directly assess hippocampal subdivisions (and thus functional units) will be helpful and necessary to reveal the specific cellular anatomy and underlying mechanisms of the observed group-by-sex interactions and sex-divergent effects. Along these lines, future studies in sufficiently powered samples may also want to follow up on the seemingly sex-specific laterality effects by testing statistically for a significant sex-by-group interaction that varies across hemispheres. At the same time, it will be desirable to extend the array of targeted brain structures beyond the hippocampus altogether, eventually complementing measures of brain structure with measures of brain function, cognition, behavior and/or psychological constructs. Moreover, while cross-sectional analyses are an excellent starting point for exposing links between meditation and brain structure, longitudinal studies with biological sex as a moderator variable will be necessary to determine the relative (and perhaps sex-specific) contribution of nature and nurture to altered brain dimensions in male and female meditators. Altogether, this will not only add to a growing body of literature suggesting a sex-divergent brain organization, but also broaden our horizons concerning sex-specific links between meditation and cerebral features as well as cognitive and behavioral parameters—perhaps even clinical outcome measures when taking meditation or mindfulness practices into patient populations (Sequeira and Ahmed, 2012; Clayton and Collins, 2014; Newberg et al., 2014).

### Conflict of Interest Statement

The authors declare that the research was conducted in the absence of any commercial or financial relationships that could be construed as a potential conflict of interest.

## Footnotes

1 http://www.loni.usc.edu/ICBM/

2 http://www.fil.ion.ucl.ac.uk/spm

3 http://dbm.neuro.uni-jena.de/vbm.html
